# Imaging corticospinal tract connectivity in injured rat spinal cord using manganese-enhanced MRI

**DOI:** 10.1186/1471-2342-6-15

**Published:** 2006-11-17

**Authors:** Mehmet Bilgen

**Affiliations:** 1 High Field MRI Research Laboratory, The University of Kansas Medical Center, 3901 Rainbow Blvd, Kansas City, KS 66160, USA; 2 Hoglund Brain Imaging Center, The University of Kansas Medical Center, 3901 Rainbow Blvd, Kansas City, KS 66160, USA; 3 Molecular and Integrative Physiology, The University of Kansas Medical Center, 3901 Rainbow Blvd, Kansas City, KS 66160, USA

## Abstract

**Background:**

Manganese-enhanced MRI (MEI) offers a novel neuroimaging modality to trace corticospinal tract (CST) in live animals. This paper expands this capability further and tests the utility of MEI to image axonal fiber connectivity in CST of injured spinal cord (SC).

**Methods:**

A rat was injured at the thoracic T4 level of the SC. The CST was labeled with manganese (Mn) injected intracortically at two weeks post injury. Next day, the injured SC was imaged using MEI and diffusion tensor imaging (DTI) modalities.

**Results:**

In vivo MEI data obtained from cervical SC confirmed that CST was successfully labeled with Mn. Ex vivo MEI data obtained from excised SC depicted Mn labeling of the CST in SC sections caudal to the lesion, which meant that Mn was transported through the injury, possibly mediated by viable CST fibers present at the injury site. Examining the ex vivo data from the injury epicenter closely revealed a thin strip of signal enhancement located ventrally between the dorsal horns. This enhancement was presumably associated with the Mn accumulation in these intact fibers projecting caudally as part of the CST. Additional measurements with DTI supported this view.

**Conclusion:**

Combining these preliminary results collectively demonstrated the feasibility of imaging fiber connectivity in experimentally injured SC using MEI. This approach may play important role in future investigations aimed at understanding the neuroplasticity in experimental SCI research.

## Background

Spinal cord injury (SCI) disrupts the functional integrity of neuronal circuits in the ascending and descending pathways in spinal cord (SC), thereby compromising the voluntary motor control of muscles below the site of the injury and the conduction of sensory signals from distal inputs. In SCI research, a particular pathway of interest is the corticospinal tract (CST) as it is the major long descending output connecting the cerebral cortex with the motor neurons in the SC [1]. Because, many different types of movements are controlled through the fibers of the CST, understanding how this tract adapts to SCI is the focus of current experimental efforts [2,3]. A number of methods have been developed to anatomically trace the CST fibers using different neuronal tracers and tract tracing procedures [4,5]. However, these conventional methods require elaborate tissue analysis based on histology and immunohistochemistry, and therefore preclude repeated measurements from the same animal. One strategy to reduce the number of post mortem procedure is the use of a transgenic animal model that has recently been engineered to specifically and completely label the CST fibers by yellow fluorescent protein [6]. Although, this novel development simplifies the work and accelerates the assessment of the regeneration and remodeling of the neuronal connections in the injured SC, it still requires invasive post mortem examination, making it unfit for dynamic imaging of live preparations.

As an alternative method, we have shown that manganese-enhanced magnetic resonance imaging (MEI) can offer new possibilities in probing the SC in live animals [7,8]. MEI is an experimental technique that uses the paramagnetic manganese ion (Mn^2+^) as a contrast agent. With an ionic radius similar to that of calcium-ion, Mn can enter cells through the voltage-gated calcium channels, and then remain confined to the intracellular compartment of intact axons. After administering Mn to the biological system, uptake and transport in the tissue of interest can be monitored remotely using in vivo magnetic resonance imaging protocols sensitive to Mn-induced altered relaxation characteristics. Applying Mn directly into the SC just rostral to a hemisectioned SC, we have demonstrated that MEI can potentially play an important role in mapping viable neuronal tissue at and below the site of injury because functional tissue on the uninjured side takes up Mn and hence is delineated on the MEI [7]. Moreover, after intracortical administration of Mn into the motor areas of the brain and stimulating the cortex electrically, we have produced robust and detectable anterograde Mn labeling of the CST from cortex caudally to the thoracic SC levels [8]. The aim of the current study is determine whether MEI can detect CST connectivity in partially injured SC. We used a rat animal model of mild thoracic SCI. This model produces an incomplete injury with functionally intact CST fibers remaining at the injury site [9]. We now demonstrate that these fibers provide an environment for transporting Mn caudal to the lesion. Visualization of the Mn-labeled tract below the lesion using MEI therefore provides a method to detect the fibers projecting through the injured section and hence fiber connectivity in injured SC.

## Methods

The experiments were conducted on one Sprague-Dawley rat (~300 g) under a protocol approved by the University of Kansas Medical Center Institutional Animal Care and Use Committee. We followed established procedures described previously for the surgery, SCI, Mn delivery and MEI scans [8-10]. After SCI, the rat was left to recover for two weeks. On post injury day 14, Mn was delivered intracortically and the motor cortex was stimulated electrically. The next day, MEI scans were performed on the living animal to confirm that the CST labeling in the SC was successful, and on excised cords to obtain high resolution MEI data around the lesion. Here, we briefly summarize these procedures and give the details of the individual components critical to the current work.

### Spinal Cord Injury (SCI)

Image artifacts from heart motion obscure the 3D visualization of the SCI at levels below the thoracic T4 level when the signal is acquired using volume coil. Therefore, SCI was induced at the T4 level to minimize these artifacts by following the injury protocol described elsewhere [9]. Briefly, the rat was anesthetized using spontaneous inhalation of 4% isoflurane for induction and maintained on a mixture of 1.5% isoflurane, 30% oxygen, and air delivered through a nose mask. A rectangular area was shaved on the back and an incision was made to expose the posterior elements of the spine. Then, a rongeur was used to perform a laminectomy at T4 to expose the spinal cord, but to leave the dura intact. After stabilizing the SC with two forceps attached to the rostral T3 and caudal T5 vertebral bodies, the laminectomized section was positioned under the impactor tip of the injury device [9]. Contusion injury was produced by using a rectangular (1 mm × 2 mm) injury tip with velocity of 1.5 m/s and contact duration of 80 ms. The deformation depth was set to 0.5 mm for producing mild partial injury with good prognosis for behavioral improvement. Next, the skin was closed and the animal was placed in a heated cage to maintain the body temperature while recovering.

### Intracortical Mn delivery

Intracortical delivery of Mn was achieved as described in [8]. On post injury day 14, the injured rat was anesthetized again using ketamine hydrochloride delivered intramuscularly at an initial dose of 150 mg/kg, followed by additional injections at doses of 5–20 mg/kg, as needed. The head of the anesthetized rat was fixed in a stereotaxic frame (Kopf Instruments, Tujunga, CA). A midline incision was made on the scalp from approximately 2.5 mm rostral to 7.5 mm caudal to the bregma, and the skin was retracted with hemostats. Bilateral 1.0 mm diameter burr holes were drilled into the skull 1.5 mm rostral to the bregma and 2.0 mm lateral to the midline using a 1 mm diameter trephine bit attached to a dental drill. An additional craniotomy was performed on one side of the skull at a location 6.5 mm caudal to the bregma and 2.0 mm lateral to the midline. Through this opening, a 2.0 mm diameter titanium screw was inserted until it rested on the dura. This screw served as the reference electrode for the electrical stimulation.

Using 1 M MnCl_2 _in a 1 μL Hamilton syringe with a tapered, graduated micropipette tip, a direct stereotaxical injection was made to deliver a total of 0.2 μL of this solution focally at 0.5 mm below the surface of the cortex through the center of the first burr hole. The solution was injected slowly over a period of five minutes. To prevent backflow, the pipette was left in place for another five minutes prior to withdrawal. This procedure was repeated for the contra lateral motor area through the second burr hole.

### Cortical stimulation

Following the Mn delivery, electrical stimulation of the cortex was achieved with a 1 mm diameter stainless steel electrode and the titanium screw serving as the reference. The electrode was connected to a DS7 Digitimer constant current stimulator (Digitimer Ltd., Hertfordshire, England) and lowered through the first burr hole on the same side as the screw until it touched to dura. A Grass S48 stimulator (Grass Medical Instruments, Quincy, MA) generated the stimulus pulse sequence used to trigger the constant current stimulator. Trains of biphasic stimuli (each phase 0.2 ms, negative first) were applied at 100 Hz for an "on" period of 5 seconds followed by an "off" period of 5 seconds. The current was adjusted until an evoked visible motor response was produced in the forelimb, hindlimb or tail, and applied for 90 minutes. If the strength of the evoked movement declined noticeably, frequency and current values were increased to maintain a constant motor response. These procedures were then repeated for the opposite cortex through a second burr hole. Immediately after stimulation, the electrodes were removed, and the skin was sutured tightly. The animal was then left to recover in its cage.

### Magnetic resonance imaging

The paramagnetic nature of Mn changes the MR properties of the tissue where it is accumulated. In particular, signals from these regions are enhanced on conventional T1-weighted spin echo (SE) images. The CST in rat SC is anatomically located in the ventral-most part of the dorsal funiculus of the SC, i.e., near the central canal between the dorsal horns of the gray matter (GM). Because of this topological arrangement, Mn-labeled CST becomes difficult to differentiate from the GM on the SE image since both structures exhibit similar intensity [8]. The use of the 3D gradient-echo (GE) sequence with short repetition time however overcomes this limitation and produces robust and detectible Mn-labeled CST in SC. More recently, inversion recovery SE (IR-SE) acquisitions were shown to offer better sensitivity to Mn in neuronal tissue [11]. Previously, we have used IR-SE imaging to demonstrate quantitatively that the T1-relaxation times of the GM and white matter (WM) are indeed slightly different in rat SC [10]. Based on the promise that IR-SE provides richer contrast enhancement and our experience with this sequence, we also performed IR-SE imaging to demonstrate its capabilities in visualizing the Mn-labeled CST in addition to the 3D GE imaging.

The MRI scans were performed 24 hr after the Mn-delivery and electrical stimulation. The rat was anesthetized using spontaneous inhalation of 4% isoflurane for induction, followed by a mixture of 1.5% isoflurane, 30% oxygen, and air delivered through a nose mask. The head was stabilized on a Plexiglas holder and positioned in a 6 cm inner diameter volume coil for MRI scanning on a 9.4 T horizontal Varian scanner (Varian Inc., Palo Alto, CA). While in the scanner, the physiological condition of the rat was monitored using ECG, respiratory and temperature probes connected to a MR-compatible small animal monitoring and gating system (Model 1025, SA Instruments, Inc., Stony Brook, NY). The body temperature was maintained at 37°C by circulating warm air with 40 % humidity using a 5 cm diameter plastic tube fitted to the back door of the magnet bore.

After confirming the placement of the animal in the magnet's isocenter with scout images, *T*_1_-weighted volumetric images covering the brain and SC at the cervical and thoracic levels were acquired using a 3D GE sequence (*T*_R_/*T*_E _= 45/4 ms and flip angle (FA) = 45°. The data were sampled on a matrix = 128 × 128 × 64 ranging over a volume of 70 × 32 × 32 mm^3^, and processed and interpolated to 256 × 256 × 128 pixels for the final display. Maximum intensity projections were generated to delineate the Mn enhancement in the CST over a desired thickness in the sagittal plane. Then, a sagittal IR-MEI was acquired (*T*_R_/*T*_E_/*T*_*I *_= 2000/12/550 ms, field-of-view (FOV) = 70 × 32 mm^2^, image matrix = 256 × 128, slice thickness = 2 mm and NEX = 4). Finally, axial IR-MEI was acquired (*T*_R_/*T*_E_/*T*_*I *_= 2000/17/550 ms, field-of-view (FOV) = 22 × 22 mm^2^, image matrix = 128 × 128, slice thickness = 2 mm and NEX = 4). The rat was then removed from the scanner, euthanized using cardiac puncture and the vertebral body was dissected from the animal. The excised sample with intact spine was scanned ex vivo at room temperature using an inductively coupled surface coil centered at the injury epicenter [10,12]. High resolution multi-slice SE images were acquired in sagittal and axial planes (sagittal parameters: *T*_R_/*T*_E _= 2500/12 ms, field-of-view = 32 × 10 mm^2^, image matrix = 256 × 128, slice thickness = 0.5 mm and NEX = 2; axial parameters: *T*_R_/*T*_E _= 2500/12 ms, field-of-view = 10 × 10 mm^2^, image matrix = 128 × 128, slice thickness = 2 mm and NEX = 2). Finally, axial IR-MEI of the excised spine and SC were acquired (*T*_R_/*T*_E_/*T*_*I *_= 2000/15/550 ms, field-of-view (FOV) = 10 × 10 mm^2^, image matrix = 128 × 128, slice thickness = 2 mm and NEX = 4).

## Results and discussion

**Figure 1:**
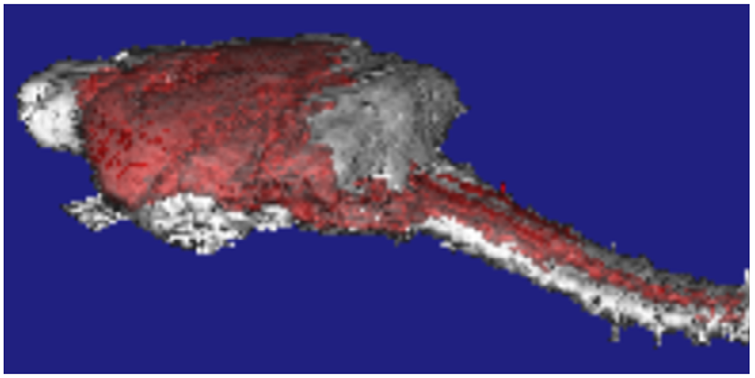
Three-dimensional visualization of the rat central nervous system and projection of the corticospinal tract (CST) pathway. The data were acquired using 3D GE-MEI after 24 h of intracortical injection of Mn and electrical stimulation of the motor cortex. The red color denotes the Mn-enhancement, labeling the CST in brain and spinal cord.

**Figure 2:**
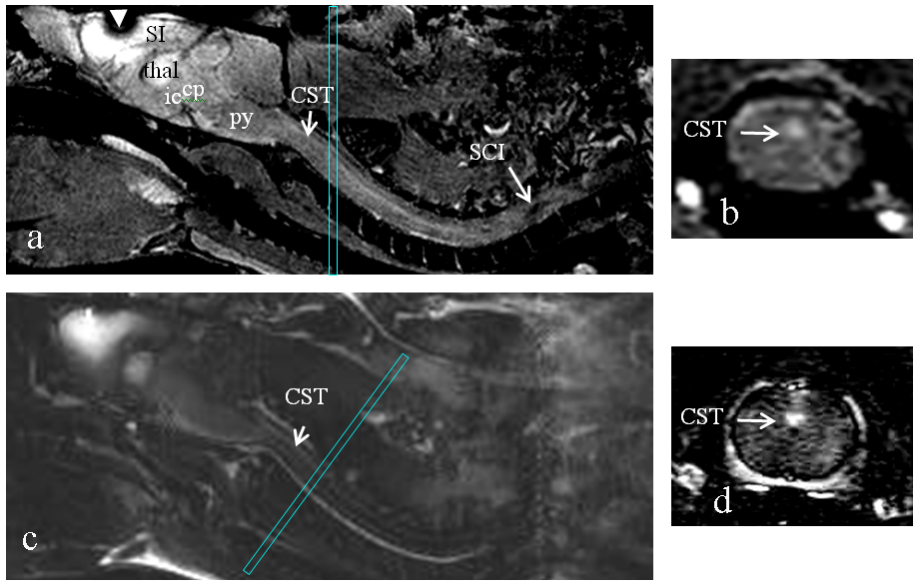
In vivo visualization of the rat central nervous system and cross sectional views of the CST pathway in the sagittal and axial planes. This data were acquired using 3D GE-MEI (a and b) and IR-MEI (c and d). The arrow labeled "SCI" points to the lesion at the T4 level. Thin-rectangles overlaid on the sagittal images represent the slice orientation for the axial images. Arrowhead in a denotes the site of the Mn injection in the brain, where the signal hypointensity is due to the presence of high local concentration of Mn. SI – primary somatosensory cortex, ic – internal capsule, thal – thalamus, cp – cerebral peduncle and py – pyramidal tract.

The CST in rat runs caudally from cortex through internal capsule, cerebral peduncle, longitudinal pontine fasciculus, pyramid, pyramidal decussation, and descends in the dorsal fasciculus of the SC [13]. Figure 1 visualizes the spatial projection of this tract in relation to the overall central nervous system by volume rendering of the acquired GE-MEI data in 3D. The Mn-enhancement in the figure is represented in red. Figure 2 depicts the Mn-enhancement in 2D views. The image in Fig. 2a shows a single sagittal slice depicting the Mn injection site, Mn-labeled CST centered within the SC and lesion as delineated by a single patch of signal hypointensity. Figure 2b shows the transverse section of the cervical SC from the imaging plane marked with a thin blue rectangle on the sagittal image. This image shows a nearly circular region of signal hyperintensity located centrally within the SC. This bright region with a well-defined boundary represents the CST labeled with Mn. The corresponding data in Figs. 2c and 2d were acquired with IR-MEI and show the capability of this approach to produce in vivo signal contrast delineating the CST. The lesion was however difficult to visualize on the IR-MEI in sagittal view. Nevertheless, these volumetric data confirmed that the above experimental procedures were successful in terms of labeling the CST with Mn in live animal.

**Figure 3:**
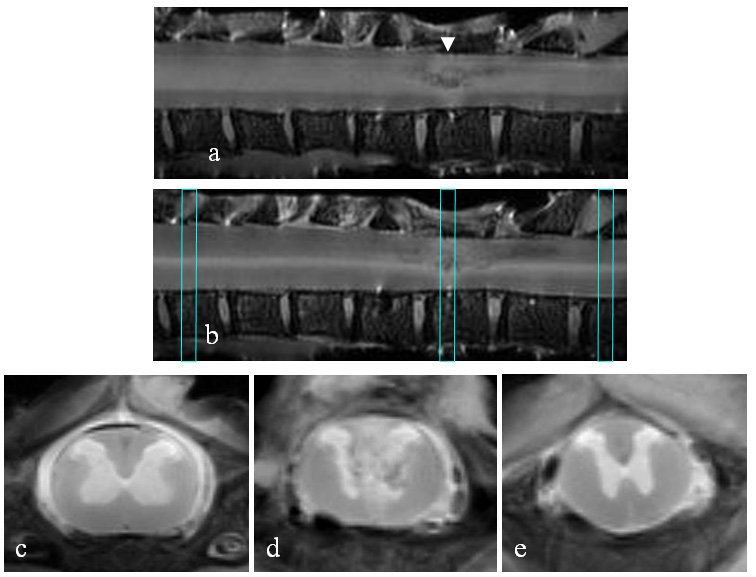
a, b) Ex vivo spin echo images showing the injury (arrow head) in an excised SC but intact spine from rostral (left) to caudal (right) direction in sagittal planes. c, d, e) Ex vivo anatomical axial spin echo images of the spinal cord from the three slice locations (c – rostral to injury, d – injury epicenter and e – caudal to injury) depicted by the thin-rectangles overlaid on the sagittal image in b.

**Figure 4:**
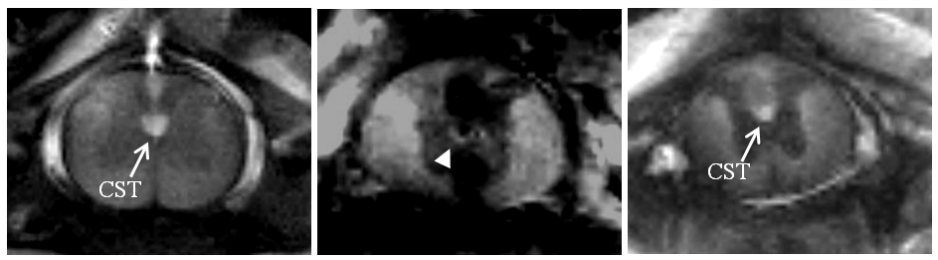
Ex vivo IR-MEIs from the same slice orientations as those images in Figs. 3-c, d and e. a) Mn-enhanced CST rostral to the injury. b) Mn-enhancement at the epicenter of the injury. c) Mn-labelling of the CST caudal to the injury. The partial signal enhancement, depicted by the arrowhead in b, is likely to represent a portion of the CST that is populated with intact fibers. By continuously projecting through the injury site, these intact fibers transport Mn from rostral to caudal sections, as indicated by the presence of focal signal enhancement in c.

After this confirmation, the experiment was continued with scans on the excised cord to get high resolution data. The resulting ex vivo images from this effort are shown in Fig. 3. The sagittal views in Figs. 3a and 3b show the injury and its extent along the SC. The anatomical images from the selected axial planes (blue rectangles in Fig. 3b) shown in Figs. 3c,d, and 3e depict the injured tissue morphology in greater detail at the epicenter as well as the normal cord at sections rostral and caudal to the injury site. Figure 4 shows the IR-MEIs acquired from the same axial slice positions. The GM on these images appears relatively darker compared to the WM in normal sections of the cord. In Fig. 4a, the pattern of signal enhancement rostral to the injury can be seen as confined spatially to the ventral-most part of the dorsal funiculus between the dorsal horns of the GM in sections rostral and caudal the injury. This enhanced region overlaps exactly with the expected anatomical location of the CST in rat SC [8,13]. Careful examination of the image from the injury epicenter (Fig. 4b) reveals a thin strip of signal enhancement, which is situated right posterior to the central canal between the dorsal roots. Signal enhancement is also seen in Fig. 4c at the expected CST location caudal to the injury. Interpretation of these results requires consideration of possible mechanisms that might facilitate trans-injury propagation of the Mn-dependent contrast. Likely mechanisms include axonal transport and extracellular diffusion of Mn. If the transport were by purely extracellular diffusion, we would expect that the axial extent of the enhancement region would expand gradually from rostral to caudal, perhaps eventually including the complete cord. However, we observed enhancement only in the location expected to be occupied by the fibers leading from the CST to distal portions of the cord, even in the images obtained caudal to the injury. Since the Mn enhancement remains closely confined to the location of the fibers into which it was originally administered, we speculate that this indicates a high level of axonal integrity since if the axons are compromised then Mn would be expected to "leak" to the extracellular space and ultimately diffuse away from the enhancing region. Based on these considerations, the Mn-labeling seen at the epicenter slide is consistent with the notion that some CST fibers might have maintained at least some level of connectivity across the injury.

**Figure 5:**
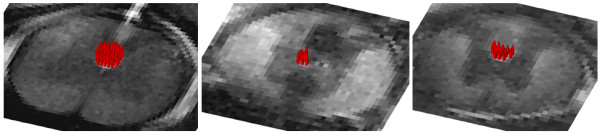
Cigar-shaped ellipsoidal representation of the principal eigenvectors estimated from the DTI measurements. The eigenvector estimates from the Mn-labeled regions were only plotted on backgrounds that are the same as those rostral, epicenter and caudal images in Fig. 4. Therefore, the density of the vectors is associated with the size of the Mn-enhancement. Vector directions are all aligned along the cord in all the three images. The direction of the alignment is consistent with the anatomical orientation of the descending neuronal fibers in the CST. The DTI data acquisition included first the baseline image, followed by the diffusion-weighted images obtained with applying diffusion sensitizing gradients along the directions (110,101,011,-110,-101,0-11). Diffusion weighting was achieved using gradient strength = 80 mT/m, width (δ) = 6.5 ms and separation (Δ) = 11 ms to produce b-value of b = 342 s/mm^2^. Other parameters were TR/TE = 2500/26 ms, FOV = 10 × 10 mm^2^, acquisition matrix = 128 × 128, slice thickness = 2 mm and NEX = 2.

To further support this interpretation, we performed diffusion tensor imaging (DTI) on the same slice locations. In previous studies of the rat optic tract, MEI and DTI have been used as complementary methods to confirm connectivity [14]. Accordingly, we expect that if the Mn-labeling at the epicenter is truly associated with the underlying connected fibers in the CST, then the water diffusion properties measured within this region would match with those obtained from above and below the lesion. Processing the acquired DTI data, pixel-by-pixel, produced estimates for the mean water diffusivity, diffusion anisotropy and diffusion direction as respectively represented by the Trace/3 (average of the diffusion tensor eigenvalues) and fractional anisotropy (FA) parameters and principal eigenvector [15]. The results from these computations are given in Table 1 and in Fig. 5. The quantitative Trace/3 and FA data in each row of the table as well as the qualitative fiber orientation data in the figure are in close agreement. The agreement between the Trace/3 values in the table meant that the water diffusivities were similar in the underlying tissues that these measurements were obtained. The agreement between the FA values indicated similar diffusion anisotropy, which indirectly suggested that the underlying tissues had axonal structure. Combining this with the observation that all the principle eigenvectors were oriented along the cord provided alternative evidence that the Mn-labeled tissue at the epicenter was part of the CST. The quantitative data in each column of the table can be seen as all comparing well. This agreement clearly provides alternative evidence that the Mn-labeled tissue at the epicenter was part of the CST. The fibers in CST are orientated parallel along the cord. These demonstrations are important because they validate our DTI measurement protocols. This agreement is very encouraging since it demonstrates the ability of two different modalities (functional MEI and structural DTI) to sensitively detect the intact fibers in the injured SC.

The use of IR imaging produces tissue contrast variations dependent on the inversion time (TI). In IR acquisitions, the slight differences between the longitudinal relaxation times (T1) in the GM and the WM (including the CST) can be utilized to visualize the GM tissue as hypointense compared to the WM and CST, unlike the image contrast typically seen in the SE images of the SC in Fig. 3[10]. The accumulation of Mn lowers the T1 in CST, introducing a new contrast behavior seen in the IR image of the SC. We determined that our acquisition parameters provided a good balance between image contrast (enhanced CST versus GM and WM signal suppression) and acquisition time at 9.4 T. Previous in vivo IR studies of the songbird brain at 7 T employed a somewhat different parameter set (*T*_R_/*T*_E_/*T*_*I *_= 4000/20/855 ms) [11]. However, we note the variations in the species and organs studied, the experimental protocols followed and the magnetic field strength, each of which contributes to the differences in imaging parameters.

It is also important to note that we have obtained high resolution images using an inductively-coupled surface coil. This coil inherently produces an inhomogeneous rf field which ultimately yields spatially variant inversion. We notice that this may be an issue in the IR-MEI acquisition and lead to an incomplete suppression of the background tissue during the inversion recovery unless the power of the 90° rf pulse is adjusted carefully to focus on the SC.

## Conclusion

This paper outlines the procedures required for successfully tracing the CST using MEI and demonstrates the potential of MEI in indirectly assessing the axonal fiber connectivity in injured rat SC. Further work is, however, warranted to confirm the reliability of tract tracing based on MEI using gold-standard histological analysis such as using transgenic [6] or anterograde tracer labeling approaches [7]. Once the veracity of the methods is confirmed, future studies aiming to address different issues such as the ability to examine the CST connectivity in more severe injuries or to explore the role of dynamic imaging of intensity build-up in the CST below the injury with time will be possible. The data from such studies may be used to assess the density and distribution of the continuous fibers or to evaluate the efficacy of promoting the fiber connectivity in injured SC with endogeneous or exogeneous interventions.

## Competing interests

The author(s) declare that they have no competing interests.

## Authors' contributions

M.B. is responsible for the conception and design of the experiments as well as the analysis and interpretation of the data.

## Pre-publication history

The pre-publication history for this paper can be accessed here:



## Figures and Tables

**Table 1:** Measurements from Mn-labeled regions, above and below the lesion, and at the epicenter of the injury.

	Above the lesion	Epicenter	Below the lesion
Trace/3 (× 10^-3 ^mm^2^/s)	0.69 ± 0.05	0.59 ± 0.07	0.64 ± 0.06
FA	0.88 ± 0.08	0.79 ± 0.13	0.85 ± 0.10

Trace/3: average of the three principal eigenvalues of the diffusion tensor. FA: fractional anisotropy index. The statistics are given as mean ± standard deviation.
